# Supplementary material for: Pharmacological Inhibition of Lipid Import and Transport Proteins in Ovarian Cancer
Source: Cancers (Basel). 2022 Dec 5;14(23):6004. doi: 10.3390/cancers14236004 (PMC9737127; doi:10.3390/cancers14236004)
Supplement: Supplementary file 1 [file cancers-14-06004-s001.zip › Supplementary Figure Legends corr TG221205.pdf]

Supplementary Figure Legends

Supplementary Figure S1. Time-dependent reduction of growth of OC cells by inhibitors of lipid handling proteins. A2780 cells were exposed for 0 – 72 hours to media containing solvent (Control) or the indicated drug concentrations before relative cell numbers (presented as optical densities) were estimated using a formazan dye assay. Means ± SD, n = 3. ANOVA followed by Scheffe test, $p < 0.001$ (***) relative to solvent-treated cells (Control).

Supplementary Figure S2. The effect of the naturally occurring LDL chelator heparin on the uptake of exogenously supplied LDLs in OC cells. A2780 and SKOV3 cells were incubated for 30 – 60 minutes with 440 µM heparin before LDL uptake was determined by the pHrodo™ Red LDL Uptake Kit as described in the Materials and Methods section. Fluorescence signals were expressed in % of untreated control. Means ± SD, n = 3. Student's t-test, $p < 0.01$ (**) and $p < 0.001$ (***) relative to untreated control.

Supplementary Figure S3. Inhibitors of lipid handling proteins do not affect the *de novo* FA synthesis in OC cells. A2780 cells were exposed to DMSO (Control) or FATP2/ARF6 inhibitor NAV2729, FABP4 inhibitor HTS01037, FABP5 inhibitor SB-FI-26, FABP4 inhibitor BMS309403, or CD36 inhibitor SSO for 48 hours before the NADPH and NADP levels were determined by a fluorometric NADP/NADPH Assay Kit as described in the Materials and Methods section. The fluorescence signals determined for NADPH were correlated with those determined for NADP, giving NADPH:NADP ratios. Fatty acid synthesis is known for its high consumption of NADPH. Therefore, the relative NADPH:NADP units obtained provide an estimate of the *de novo* FA synthesis in the cells [45]. These ratios did not significantly change upon drug treatment. Means ± SD, n = 3.

Supplementary Figure S4. Histograms and scatter plots of one of three separate experiments for determination of cell cycle distribution of untreated and treated OC cells using flow cytometry. A2780 cells were incubated in medium containing solvent (0.1% DMSO) or various concentrations of small molecule inhibitors of cellular lipid handling proteins at 37° C for 48 hours.

Supplementary Figure S5. Scatter plots of one of three separate experiments for determination of apoptosis of untreated and treated OC cells using flow cytometry. A2780 cells were incubated in medium containing solvent (0.1% DMSO), 7µM of the multi-kinase inhibitor ponatinib (positive control) [39] or various concentrations of small molecule inhibitors of cellular lipid handling proteins at 37° C for 48 hours. Cells were then examined by flow cytometry to determine the percentage of APC annexin V positive cells (A) or active caspase-3 positive cells (B). Results show relative cell numbers in percent of total cell number. The fraction of apoptotic cells included the lower right quadrants (allophycocyanin[APC] annexin V positive/propidium iodide[PI] negative cells) and upper right quadrants (APC annexin V positive/PI positive cells) of the charts, while the lower left quadrants represent viable cells (APC annexin V negative/propidium iodide[PI] negative cells) and the upper left quadrants represent all necrotic cells (APC annexin V negative/PI positive cells).

Supplementary Figure S6. Gene Ontology (Biological processes) enrichment analysis of differentially expressed genes after a 4-hours treatment of A2780 and SKOV3 cells with BMS309403, NAV2729, HTS01037, SB-FI-26 or SSO compared to DMSO controls, respectively. Drug concentrations were close to the IC_{50} values for inhibition of cell growth and uptake of exogenous FA (A2780: 6µM NAV2729, 30µM BMS309403, 20µM HTS01037, 30µM SB-FI-26, 100µM SSO; SKOV3: 10µM NAV2729, 80µM BMS309403, 80µM HTS01037, 80µM SB-FI-26, 200µM SSO). Enrichment analysis was performed in both cell lines using the compareCluster function from clusterProfiler considering a Benjamini-Hochberg adjusted p-value (FDR) < 0.05. As usual, GeneRatio denotes the ratio of the number of genes in the particular input list associated with the given GO term relative to the total number of genes in that GO term. Processes associated with stress response are written in red letters, processes associated with apoptosis are written in blue letters, other processes are written in black letters. Treatment of cells with BMS309403 and SSO for 4 hours did not result in significant changes in gene expression. Thus, no enrichment analyses were performed.

Supplementary Figure S7. Gene Ontology (Biological processes) enrichment analysis of differentially expressed genes after a 48-hours treatment of A2780 and SKOV3 cells with BMS309403, NAV2729, HTS01037, SB-FI-26 or SSO compared to DMSO controls, respectively. Drug concentrations were close to the IC_{50} values for inhibition of cell growth and uptake of exogenous FA (A2780: 6µM NAV2729, 30µM BMS309403, 20µM HTS01037, 30µM SB-FI-26, 100µM SSO; SKOV3: 10µM NAV2729, 80µM BMS309403, 80µM HTS01037, 80µM SB-FI-26, 200µM SSO). Enrichment analysis was performed in both cell lines using the compareCluster function from clusterProfiler considering a Benjamini-Hochberg adjusted p-value (FDR) < 0.05. As usual, GeneRatio denotes the ratio of the number of genes in the particular input list associated with the given GO term relative to the total number of genes in that GO term. Processes associated with cell cycle are written in red letters, processes associated with metabolic pathways are written in blue letters, other processes are written in black letters.

Supplementary Figure S8. Uncropped Western blot images shown in Figure 2. Various lipid handling proteins (FABP4, FABP5, FABP6, FATP2, ARF6, CD36, LDLR) expressed in A2780 and SKOV3 OC cells and the effects of inhibitors of these lipid handling proteins on their expression. Monolayer cultures were exposed for 48 hours with drug concentrations close to the IC_{50} values for cell growth inhibition and exogenous FA uptake as shown in Figures 1 and 3 (A2780: 7.5 µM NAV2729, 30 µM BMS309403, 22.5 µM HTS01037, 30 µM SB-FI-26, and 100 µM SSO; SKOV3: 10 µM NAV2729, 80 µM BMS309403, 80 µM HTS01037, 80 µM SB-FI-26, and 200 µM SSO). Then, cell lysates were processed by SDS-PAGE, immunoblotting, and enhanced chemiluminescence (see Materials and Methods Section). Control II was 0.2% (v/v) DMSO for SSO and Control I was 0.06% (v/v) DMSO for the other drugs. Actin served as loading control. Representative data from one of two separate experiments.
