# Supplementary material for: Pharmacological Inhibition of Lipid Import and Transport Proteins in Ovarian Cancer
Source: Cancers (Basel). 2022 Dec 5;14(23):6004. doi: 10.3390/cancers14236004 (PMC9737127; doi:10.3390/cancers14236004)

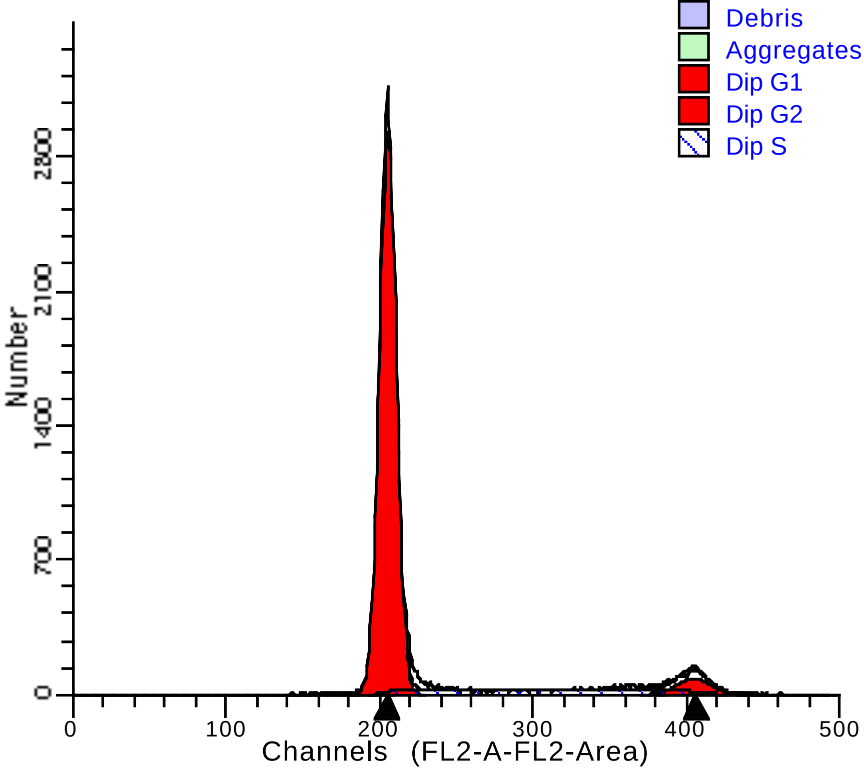

File analyzed: A2780 DMSO-Co.00
Date analyzed: 5-Feb-2019
Model: 1DA0n_DSD
Analysis type: Manual analysis

Ploidy Mode: First cycle is diploid

Diploid: 100.00 %
Dip G1: 83.91 % at 205.34
Dip G2: 4.53 % at 404.51
Dip S: 11.56 % G2/G1: 1.97
%CV: 2.67

Total S-Phase: 11.56 %
Total B.A.D.: 1.24 %

Debris: 0.20 %
Aggregates: 1.73 %
Modeled events: 48884
All cycle events: 47939
Cycle events per channel: 239
RCS: 3.429

2.67	% CV
47939	Cell Number
239	Avg. Cells Per Channel
n/a	Aneuploid Fraction
1.24	% B.A.D.
3.43	RCS

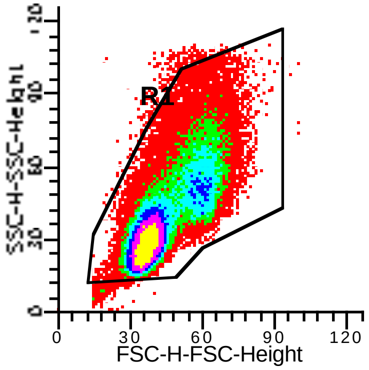

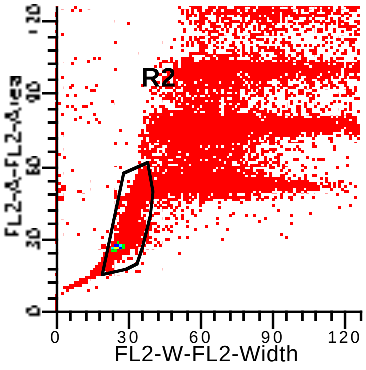

ModFitLT V3.1(PMac)

Supplementary Figure S4

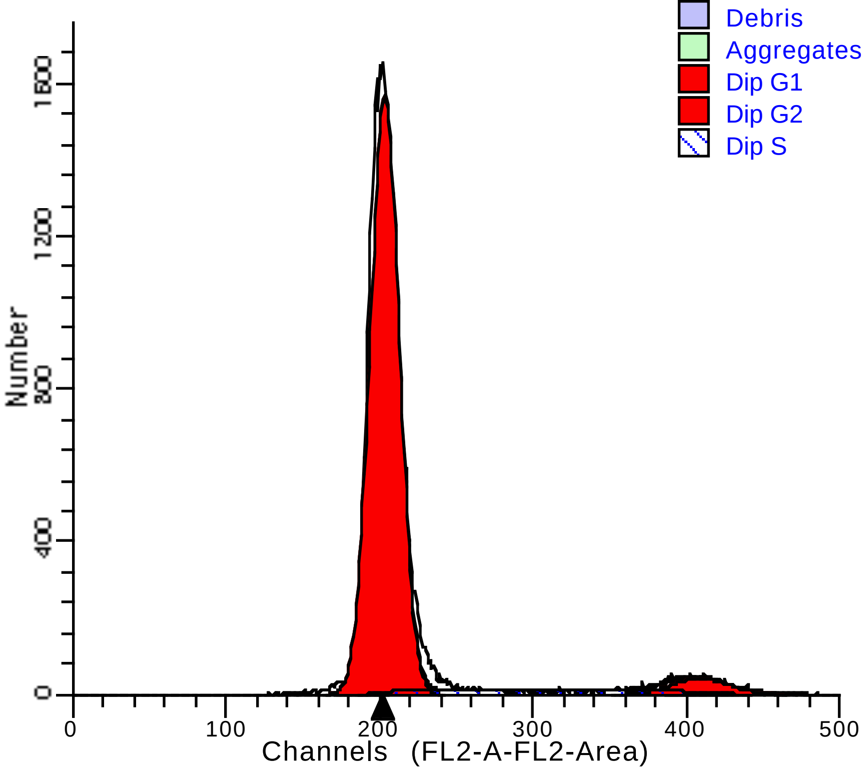

File analyzed: A2780 30uM BMS.0
Date analyzed: 5-Feb-2019
Model: 1DA0n_DSD
Analysis type: Manual analysis

Ploidy Mode: First cycle is diploid

Diploid: 100.00 %
Dip G1: 89.42 % at 203.32
Dip G2: 4.67 % at 408.67
Dip S: 5.91 % G2/G1: 2.01
%CV: 4.63

Total S-Phase: 5.91 %
Total B.A.D.: 0.10 %

Debris: 0.18 %
Aggregates: 0.13 %
Modeled events: 41408
All cycle events: 41276
Cycle events per channel: 200
RCS: 4.822

4.63	% CV
41276	Cell Number
200	Avg. Cells Per Channel
n/a	Aneuploid Fraction
0.10	% B.A.D.
4.82	RCS

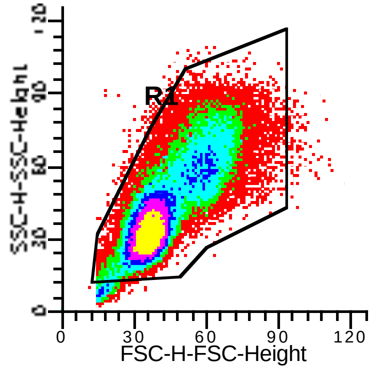

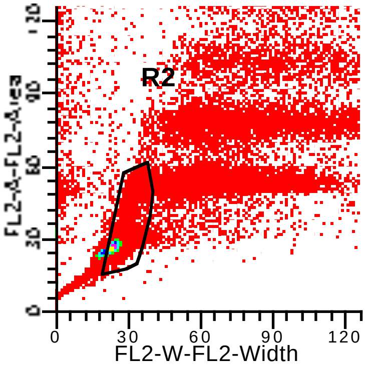

ModFitLT V3.1(PMac)

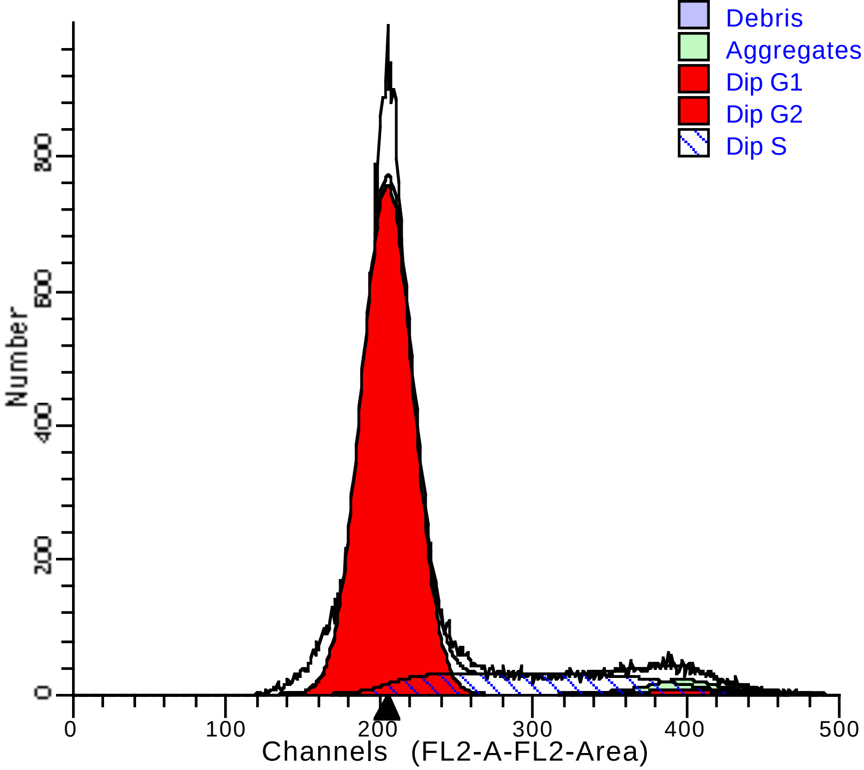

File analyzed: A2780 40uM BMS.0
Date analyzed: 5-Feb-2019
Model: 1DA0n_DSD
Analysis type: Manual analysis

Ploidy Mode: First cycle is diploid

Diploid: 100.00 %
Dip G1: 83.49 % at 204.99
Dip G2: 1.16 % at 395.63
Dip S: 15.35 % G2/G1: 1.93
%CV: 8.14

Total S-Phase: 15.35 %
Total B.A.D.: 2.66 %

Debris: 0.20 %
Aggregates: 3.56 %
Modeled events: 39413
All cycle events: 37930
Cycle events per channel: 198
RCS: 4.268

8.14	% CV
37930	Cell Number
198	Avg. Cells Per Channel
n/a	Aneuploid Fraction
2.66	% B.A.D.
4.27	RCS

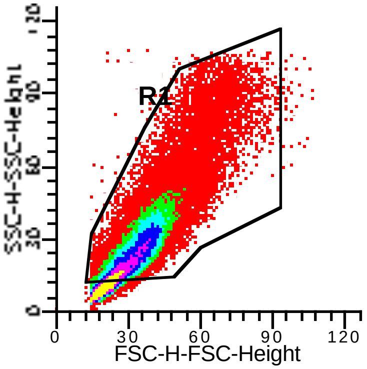

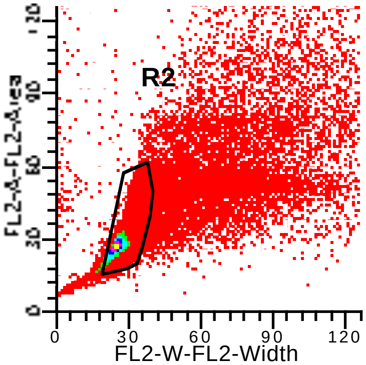

ModFitLT V3.1(PMac)

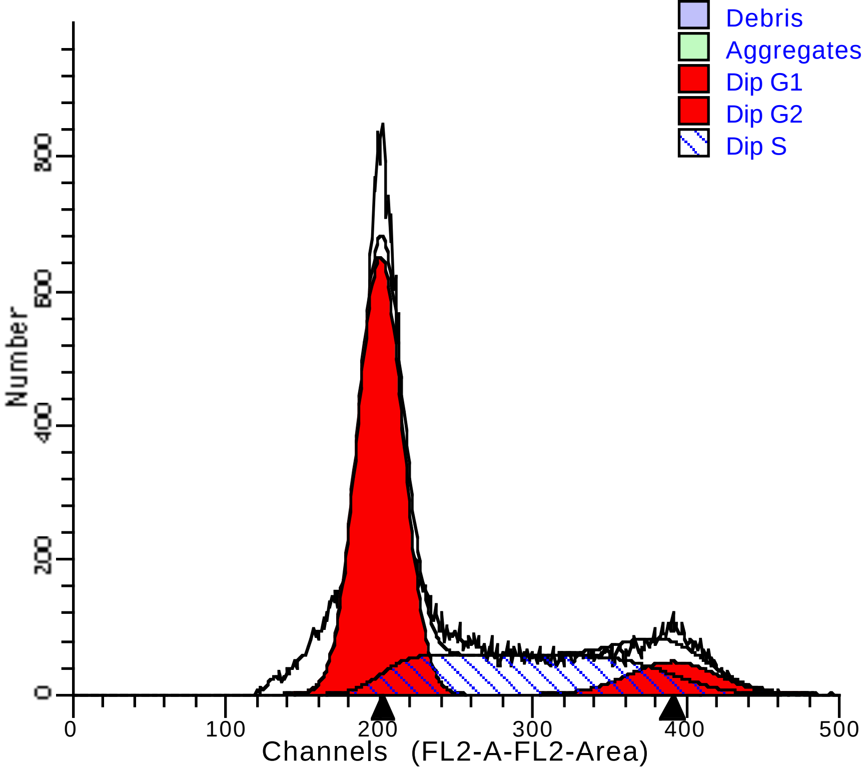

File analyzed: A2780 50uM BMS.0
Date analyzed: 5-Feb-2019
Model: 1DA0n_DSD
Analysis type: Manual analysis

Ploidy Mode: First cycle is diploid

Diploid: 100.00 %
Dip G1: 61.95 % at 200.34
Dip G2: 9.00 % at 390.67
Dip S: 29.05 % G2/G1: 1.95
%CV: 7.31

Total S-Phase: 29.05 %
Total B.A.D.: 0.06 %

Debris: 0.24 %
Aggregates: 0.13 %
Modeled events: 38768
All cycle events: 38627
Cycle events per channel: 202
RCS: 7.004

7.31	% CV
38627	Cell Number
202	Avg. Cells Per Channel
n/a	Aneuploid Fraction
0.06	% B.A.D.
7.00	RCS

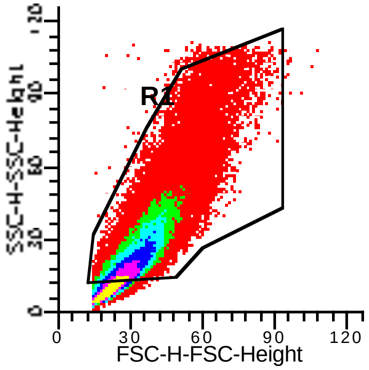

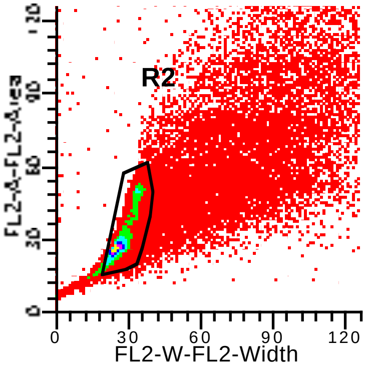

ModFitLT V3.1(PMac)

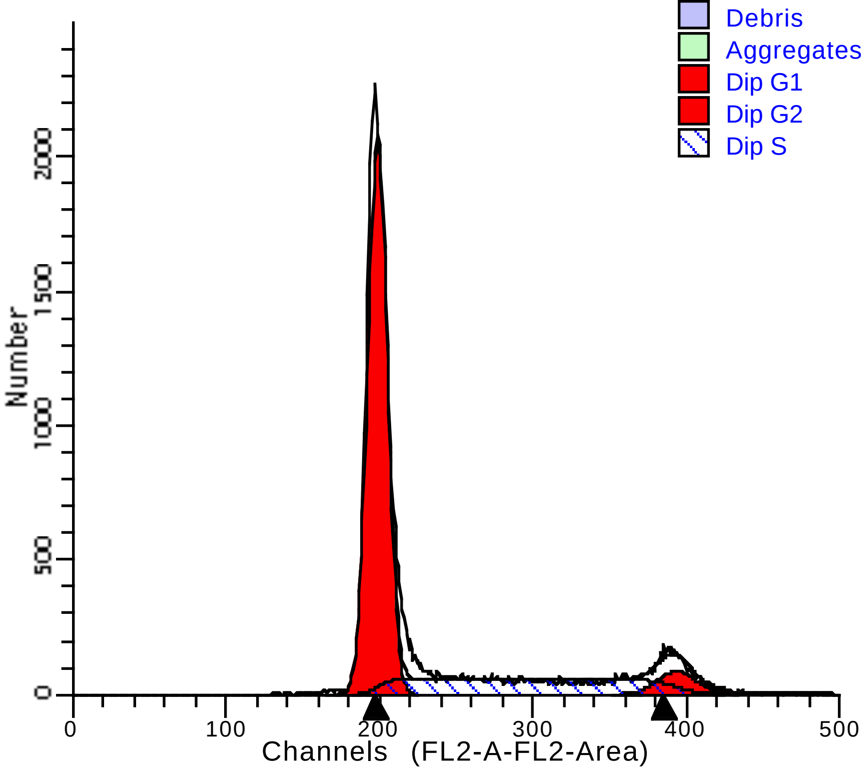

File analyzed: A2780 30uM HTS.01
Date analyzed: 5-Feb-2019
Model: 1DA0n_DSD
Analysis type: Manual analysis

Ploidy Mode: First cycle is diploid

Diploid: 100.00 %
Dip G1: 70.86 % at 198.64
Dip G2: 5.84 % at 393.30
Dip S: 23.30 % G2/G1: 1.98
%CV: 3.20

Total S-Phase: 23.30 %
Total B.A.D.: 1.39 %

Debris: 0.18 %
Aggregates: 2.41 %
Modeled events: 47491
All cycle events: 46258
Cycle events per channel: 236
RCS: 6.707

3.20	% CV
46258	Cell Number
236	Avg. Cells Per Channel
n/a	Aneuploid Fraction
1.39	% B.A.D.
6.71	RCS

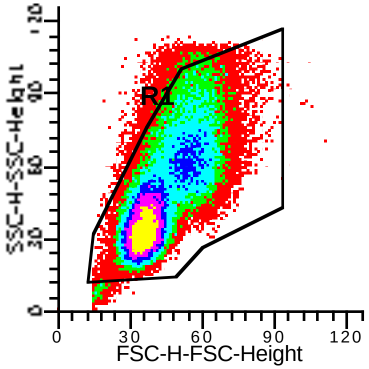

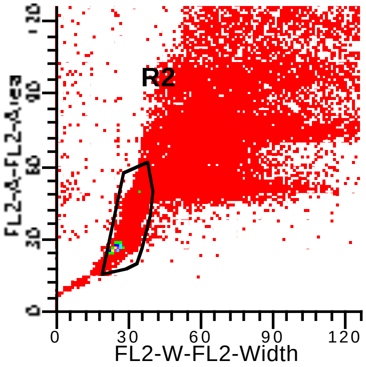

ModFitLT V3.1(PMac)

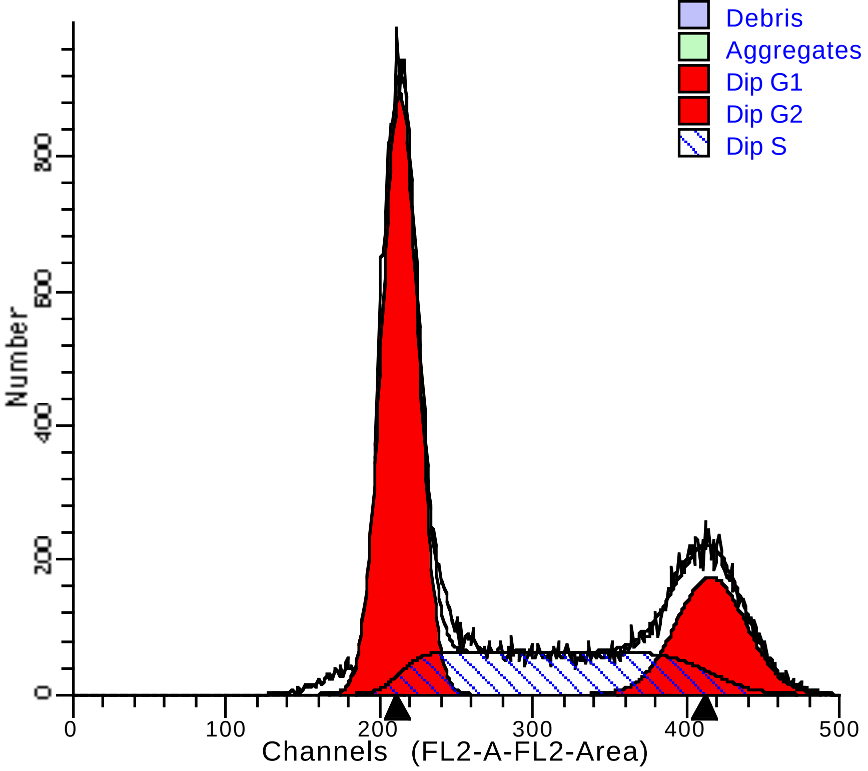

File analyzed: A2780 40uM HTS.01
Date analyzed: 5-Feb-2019
Model: 1DA0n_DSD
Analysis type: Manual analysis

Ploidy Mode: First cycle is diploid

Diploid: 100.00 %
Dip G1: 53.52 % at 213.18
Dip G2: 20.35 % at 415.71
Dip S: 26.13 % G2/G1: 1.95
%CV: 5.50

Total S-Phase: 26.13 %
Total B.A.D.: 1.07 %

Debris: 0.21 %
Aggregates: 1.64 %
Modeled events: 50027
All cycle events: 49098
Cycle events per channel: 241
RCS: 3.299

5.50	% CV
49098	Cell Number
241	Avg. Cells Per Channel
n/a	Aneuploid Fraction
1.07	% B.A.D.
3.30	RCS

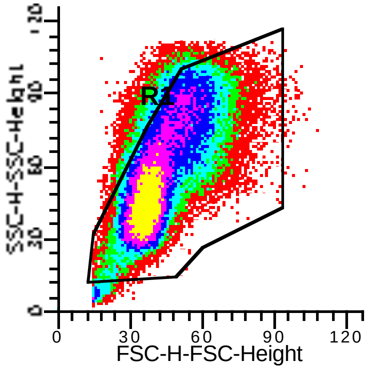

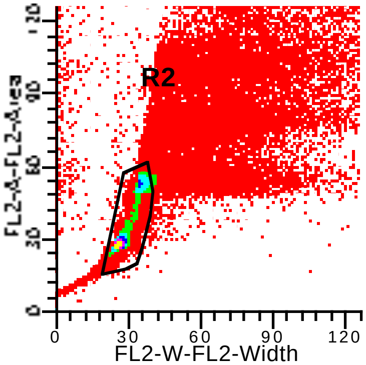

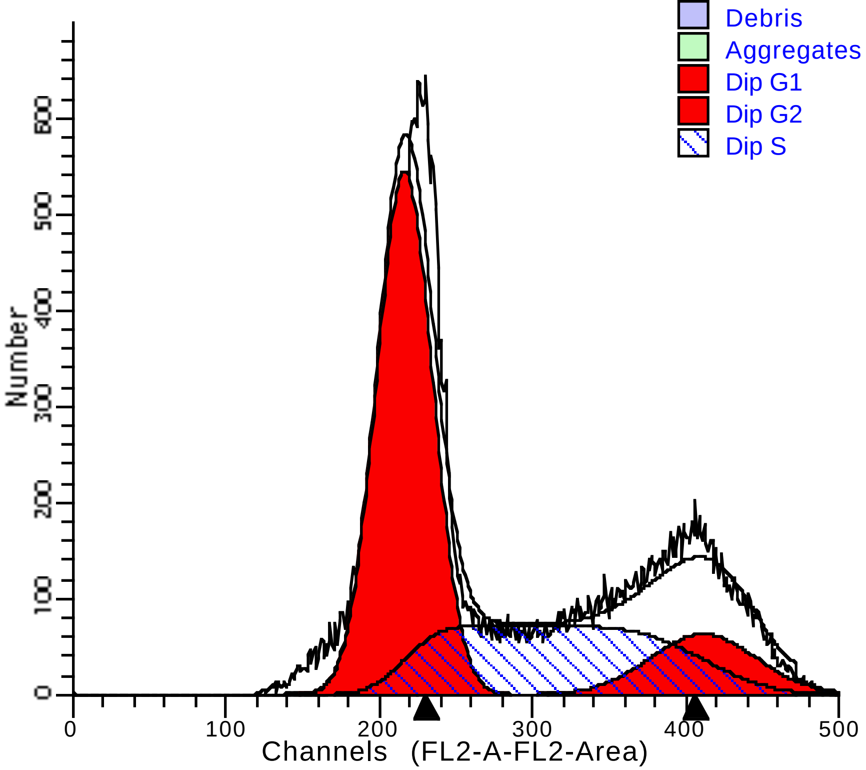

File analyzed: A2780 50uM HTS.01
Date analyzed: 5-Feb-2019
Model: 1DA0n_DSD
Analysis type: Manual analysis

Ploidy Mode: First cycle is diploid

Diploid: 100.00 %
Dip G1: 56.12 % at 216.36
Dip G2: 12.17 % at 411.09
Dip S: 31.71 % G2/G1: 1.90
%CV: 8.43

Total S-Phase: 31.71 %
Total B.A.D.: 4.49 %

Debris: 0.24 %
Aggregates: 7.29 %
Modeled events: 48030
All cycle events: 44412
Cycle events per channel: 227
RCS: 5.456

8.43	% CV
44412	Cell Number
227	Avg. Cells Per Channel
n/a	Aneuploid Fraction
4.49	% B.A.D.
5.46	RCS

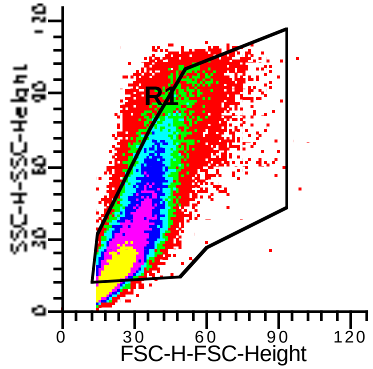

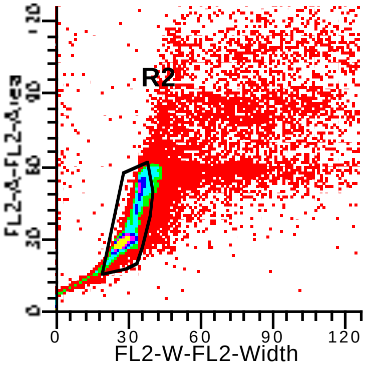

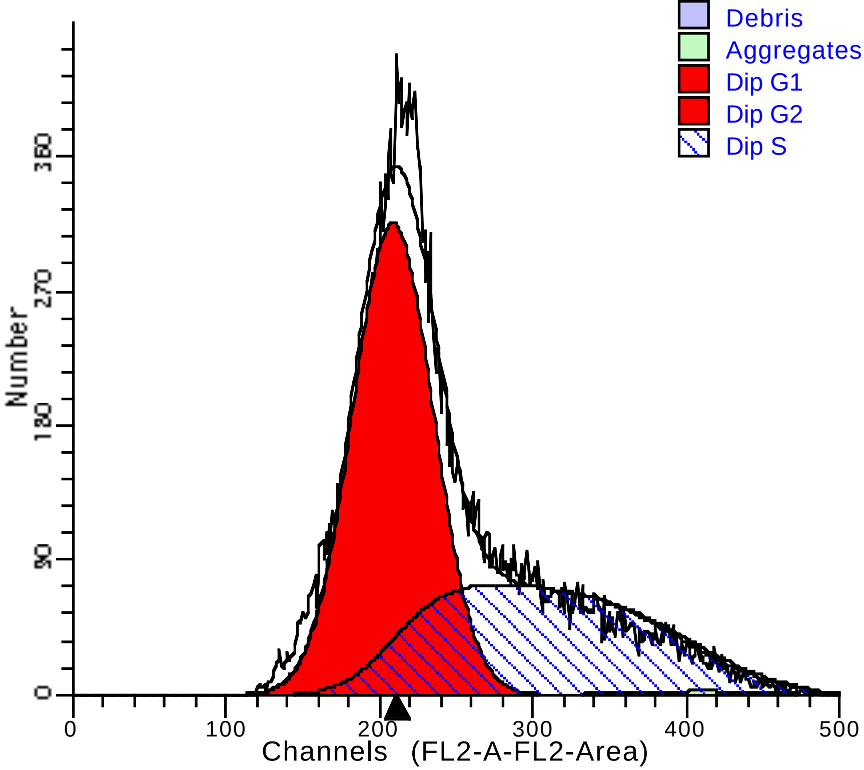

File analyzed: A2780 10uM NAV.02
Date analyzed: 5-Feb-2019
Model: 1DA0n_DSD
Analysis type: Manual analysis

Ploidy Mode: First cycle is diploid

Diploid: 100.00 %
Dip G1: 59.93 % at 208.55
Dip G2: 0.00 % at 396.25
Dip S: 40.07 % G2/G1: 1.90
%CV: 12.53

Total S-Phase: 40.07 %
Total B.A.D.: 0.40 %

Debris: 0.06 %
Aggregates: 0.74 %
Modeled events: 34733
All cycle events: 34455
Cycle events per channel: 183
RCS: 3.573

12.53	% CV
34455	Cell Number
183	Avg. Cells Per Channel
n/a	Aneuploid Fraction
0.40	% B.A.D.
3.57	RCS

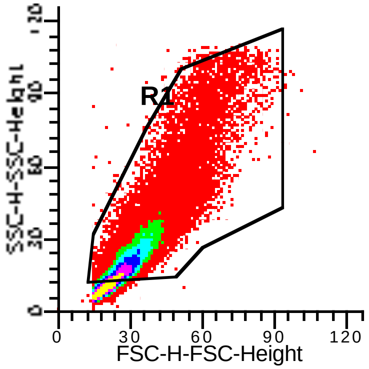

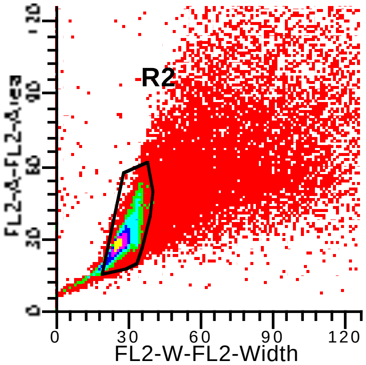

ModFitLT V3.1(PMac)

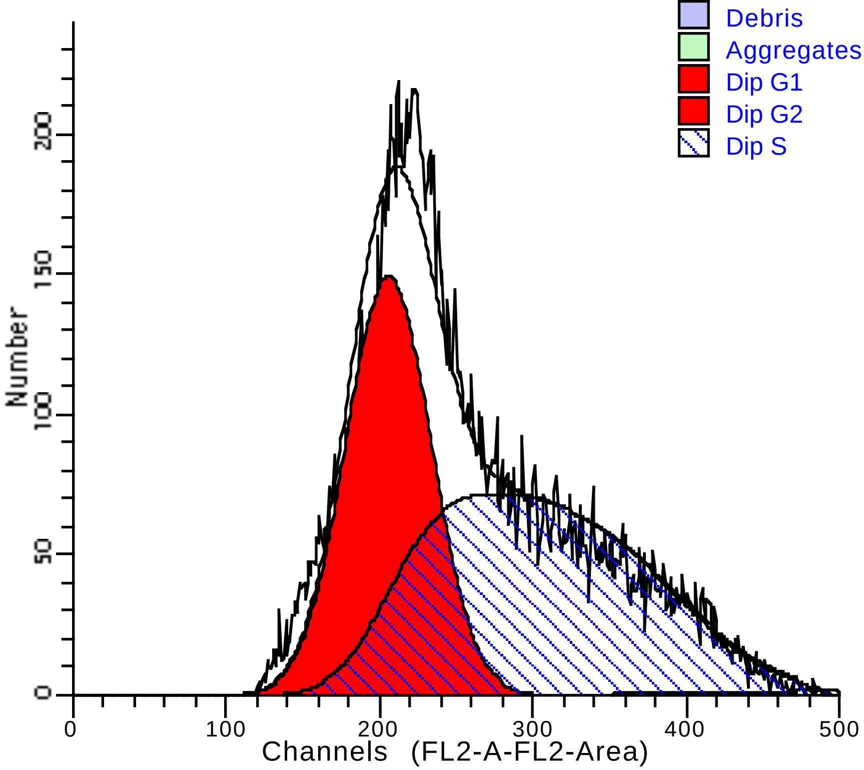

File analyzed: A2780 20uM NAV.02
Date analyzed: 5-Feb-2019
Model: 1DA0n_DSD
Analysis type: Manual analysis

Ploidy Mode: First cycle is diploid

Diploid: 100.00 %
Dip G1: 43.49 % at 205.71
Dip G2: 0.00 % at 390.85
Dip S: 56.51 % G2/G1: 1.90
%CV: 13.50

Total S-Phase: 56.51 %
Total B.A.D.: 0.23 %

Debris: 0.02 %
Aggregates: 0.57 %
Modeled events: 23994
All cycle events: 23854
Cycle events per channel: 128
RCS: 2.534

13.50	% CV
23854	Cell Number
128	Avg. Cells Per Channel
n/a	Aneuploid Fraction
0.23	% B.A.D.
2.53	RCS

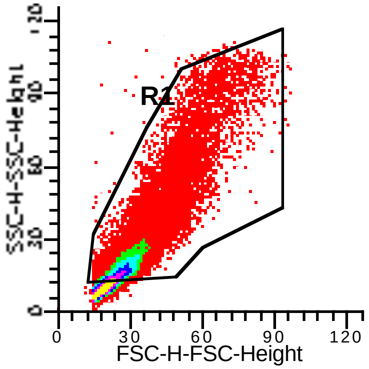

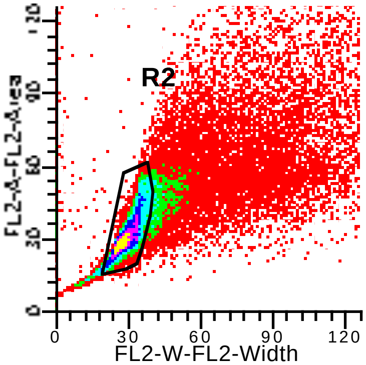

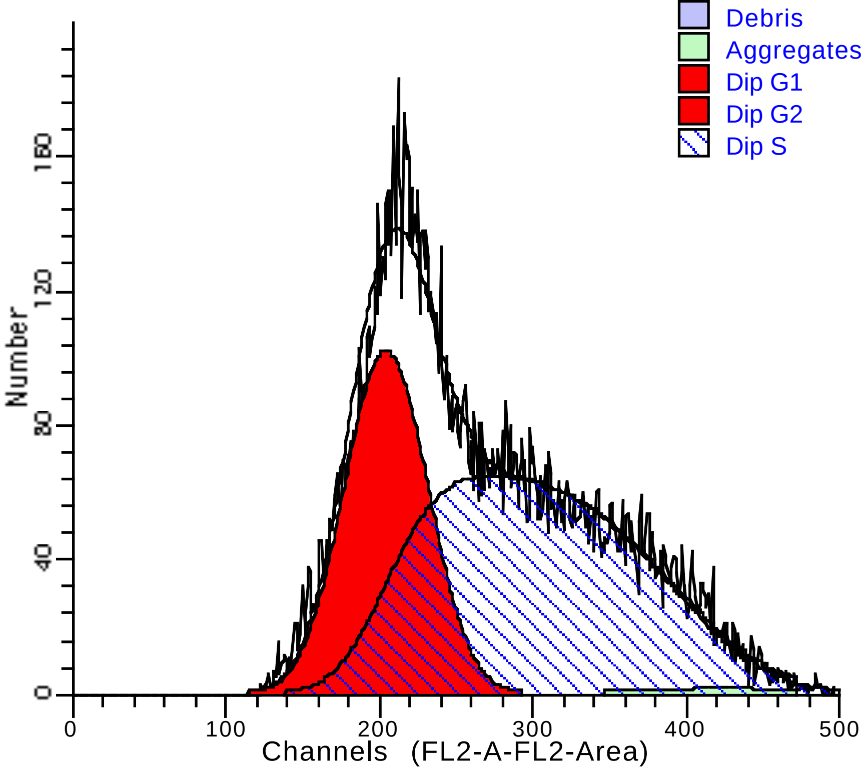

File analyzed: A2780 30uM NAV.02
Date analyzed: 5-Feb-2019
Model: 1DA0n_DSD
Analysis type: Manual analysis

Ploidy Mode: First cycle is diploid

Diploid: 100.00 %
Dip G1: 36.52 % at 204.27
Dip G2: 0.00 % at 388.11
Dip S: 63.48 % G2/G1: 1.90
%CV: 13.30

Total S-Phase: 63.48 %
Total B.A.D.: 0.30 %

Debris: 0.01 %
Aggregates: 0.89 %
Modeled events: 19279
All cycle events: 19105
Cycle events per channel: 103
RCS: 1.290

13.30	% CV
19105	Cell Number
103	Avg. Cells Per Channel
n/a	Aneuploid Fraction
0.30	% B.A.D.
1.29	RCS

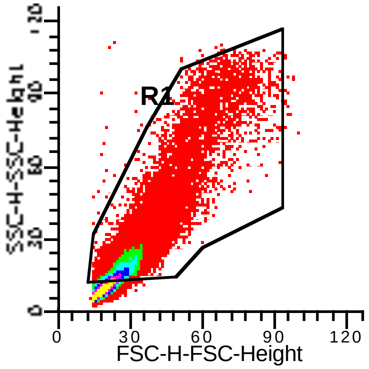

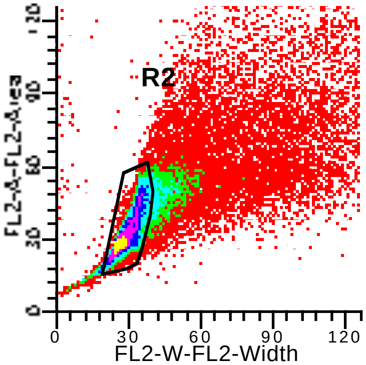

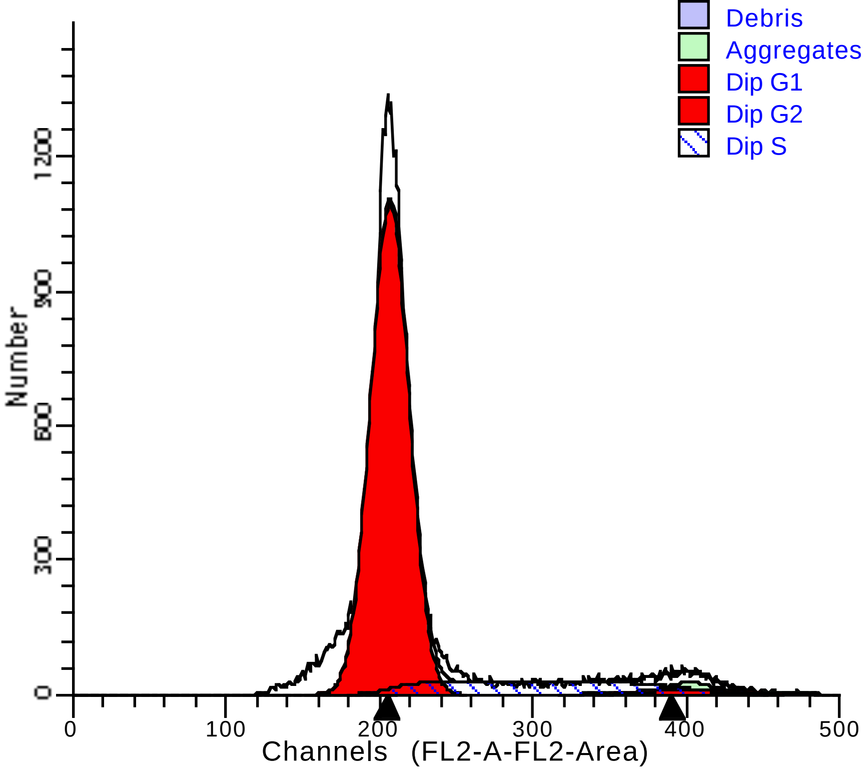

File analyzed: A2780 30uM SB-FI
Date analyzed: 5-Feb-2019
Model: 1DA0n_DSD
Analysis type: Manual analysis

Ploidy Mode: First cycle is diploid

Diploid: 100.00 %
Dip G1: 85.18 % at 206.60
Dip G2: 1.69 % at 400.81
Dip S: 13.13 % G2/G1: 1.94
%CV: 6.05

Total S-Phase: 13.13 %
Total B.A.D.: 2.81 %

Debris: 0.24 %
Aggregates: 3.40 %
Modeled events: 41773
All cycle events: 40254
Cycle events per channel: 206
RCS: 8.136

6.05	% CV
40254	Cell Number
206	Avg. Cells Per Channel
n/a	Aneuploid Fraction
2.81	% B.A.D.
8.14	RCS

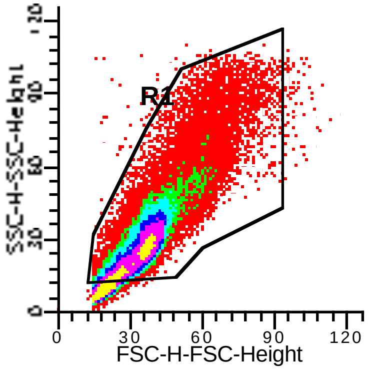

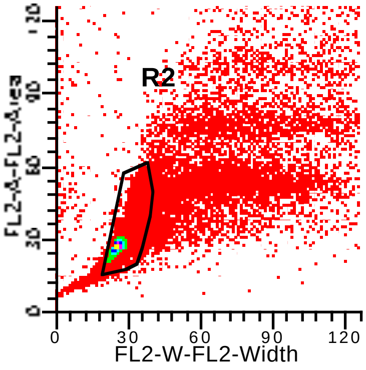

ModFitLT V3.1(PMac)

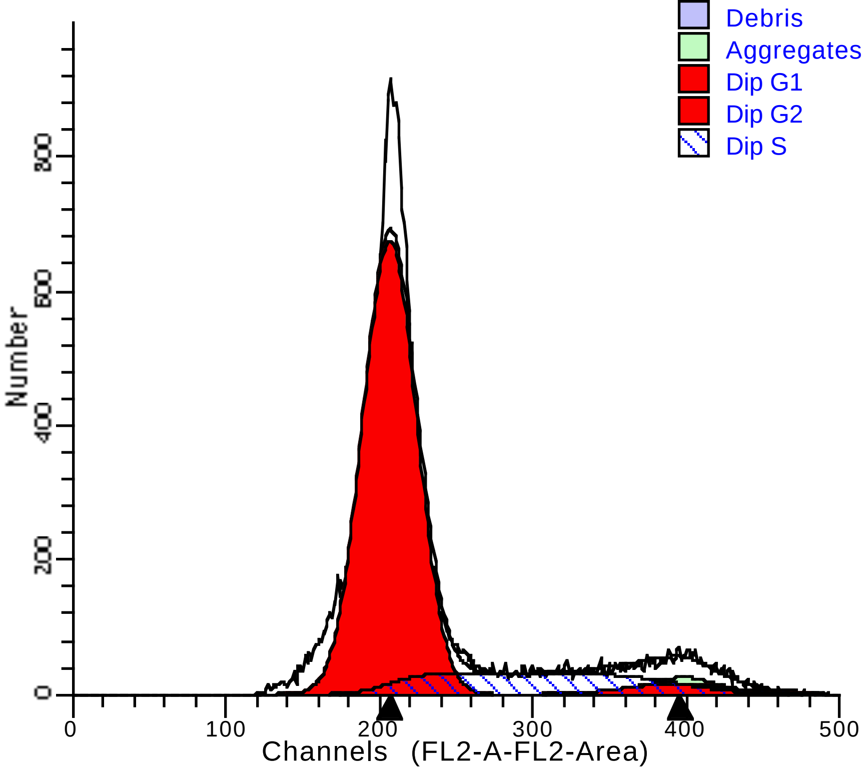

File analyzed: A2780 40uM SB-FI
Date analyzed: 5-Feb-2019
Model: 1DA0n_DSD
Analysis type: Manual analysis

Ploidy Mode: First cycle is diploid

Diploid: 100.00 %
Dip G1: 80.23 % at 206.52
Dip G2: 3.55 % at 394.45
Dip S: 16.22 % G2/G1: 1.91
%CV: 8.50

Total S-Phase: 16.22 %
Total B.A.D.: 3.21 %

Debris: 0.21 %
Aggregates: 4.28 %
Modeled events: 38796
All cycle events: 37054
Cycle events per channel: 196
RCS: 5.316

8.50	% CV
37054	Cell Number
196	Avg. Cells Per Channel
n/a	Aneuploid Fraction
3.21	% B.A.D.
5.32	RCS

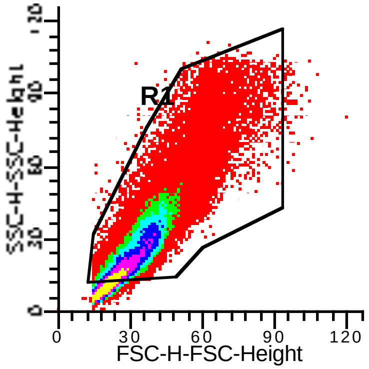

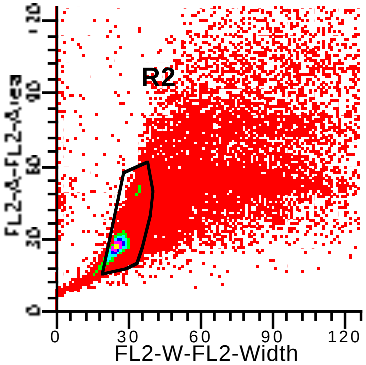

ModFitLT V3.1(PMac)

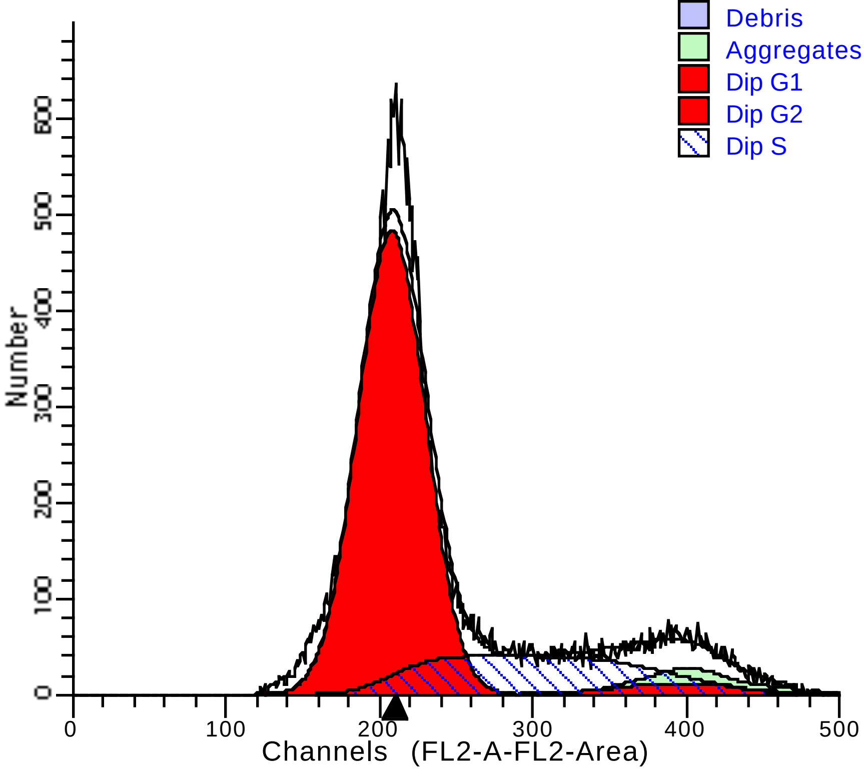

File analyzed: A2780 50uM SB-FI
Date analyzed: 5-Feb-2019
Model: 1DA0n_DSD
Analysis type: Manual analysis

Ploidy Mode: First cycle is diploid

Diploid: 100.00 %
Dip G1: 75.33 % at 207.86
Dip G2: 3.30 % at 394.94
Dip S: 21.37 % G2/G1: 1.90
%CV: 10.51

Total S-Phase: 21.37 %
Total B.A.D.: 3.98 %

Debris: 0.18 %
Aggregates: 5.76 %
Modeled events: 37342
All cycle events: 35121
Cycle events per channel: 187
RCS: 2.894

10.51	% CV
35121	Cell Number
187	Avg. Cells Per Channel
n/a	Aneuploid Fraction
3.98	% B.A.D.
2.89	RCS

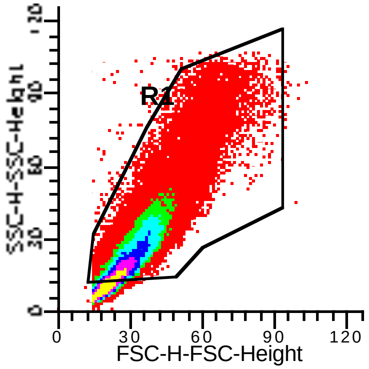

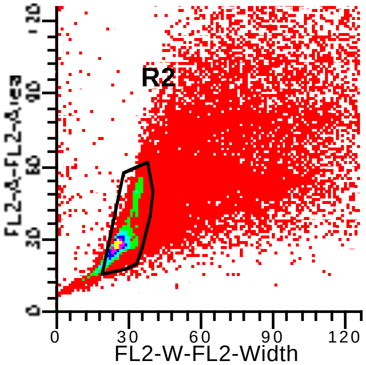

Supplement: Supplementary file 1 [file cancers-14-06004-s001.zip › Supplementary Figure S4.pdf]
