# Supplementary material for: Pharmacological Inhibition of Lipid Import and Transport Proteins in Ovarian Cancer
Source: Cancers (Basel). 2022 Dec 5;14(23):6004. doi: 10.3390/cancers14236004 (PMC9737127; doi:10.3390/cancers14236004)
Supplement: Supplementary file 1 [file cancers-14-06004-s001.zip › Supplementary Table S1.pdf]

Supplementary Table S1: Drug-induced modulation of mRNAs encoding targeted lipid handling proteins as determined by RNA-Seq analysis

	mRNA Expression (fold change of treated cells relative to untreated control cells)										
	A2780						SKOV3				
	NAV2729	BMS309403	HTS01037	SB-FI-26	SSO		NAV2729	BMS309403	HTS01037	SB-FI-26	SSO
FABP4	~	~	~	~	~	FABP4	~	~	~	~	~
FABP5	~	~	~	~	~	FABP5	~	~	~	~	~
FABP6	~	~	~	~	~	FABP6	~	~	~	~	~
FATP2	~	~	~	~	~	FATP2	0,20	0,50	0,54	~	~
ARF6	~	~	~	~	~	ARF6	~	~	~	~	~
CD36	~	~	~	~	~	CD36	~	~	~	~	~
LDLR	1,55	~	~	~	2,48	LDLR	~	~	0,65	0,51	~

~ … no change
