# Supplementary figures and images for: Pharmacological Inhibition of Lipid Import and Transport Proteins in Ovarian Cancer
Source: Cancers (Basel). 2022 Dec 5;14(23):6004. doi: 10.3390/cancers14236004 (PMC9737127; doi:10.3390/cancers14236004)

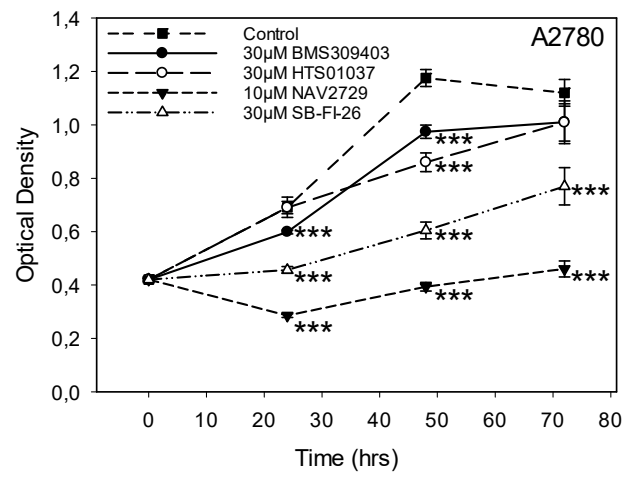

Supplementary Figure S1

Supplement: Supplementary file 1 [file cancers-14-06004-s001.zip › Supplementary Figure S1.pdf]

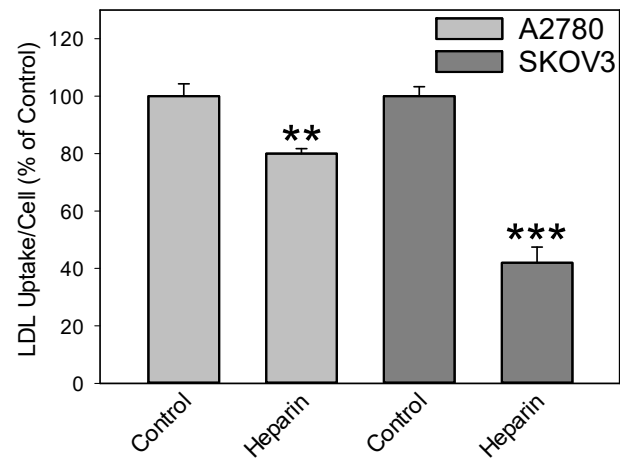

Supplement: Supplementary file 1 [file cancers-14-06004-s001.zip › Supplementary Figure S2.pdf]

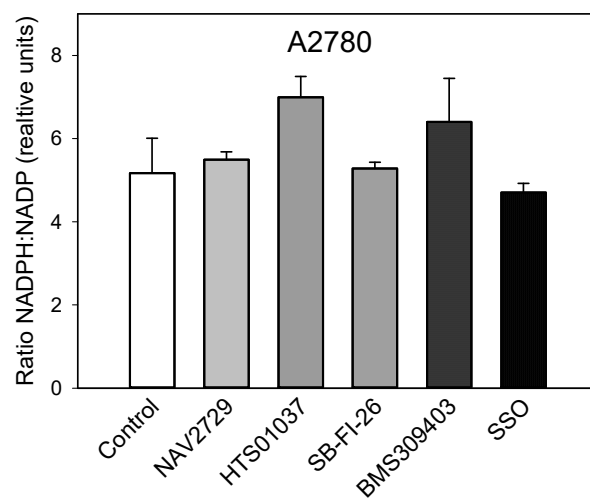

Supplement: Supplementary file 1 [file cancers-14-06004-s001.zip › Supplementary Figure S3.pdf]

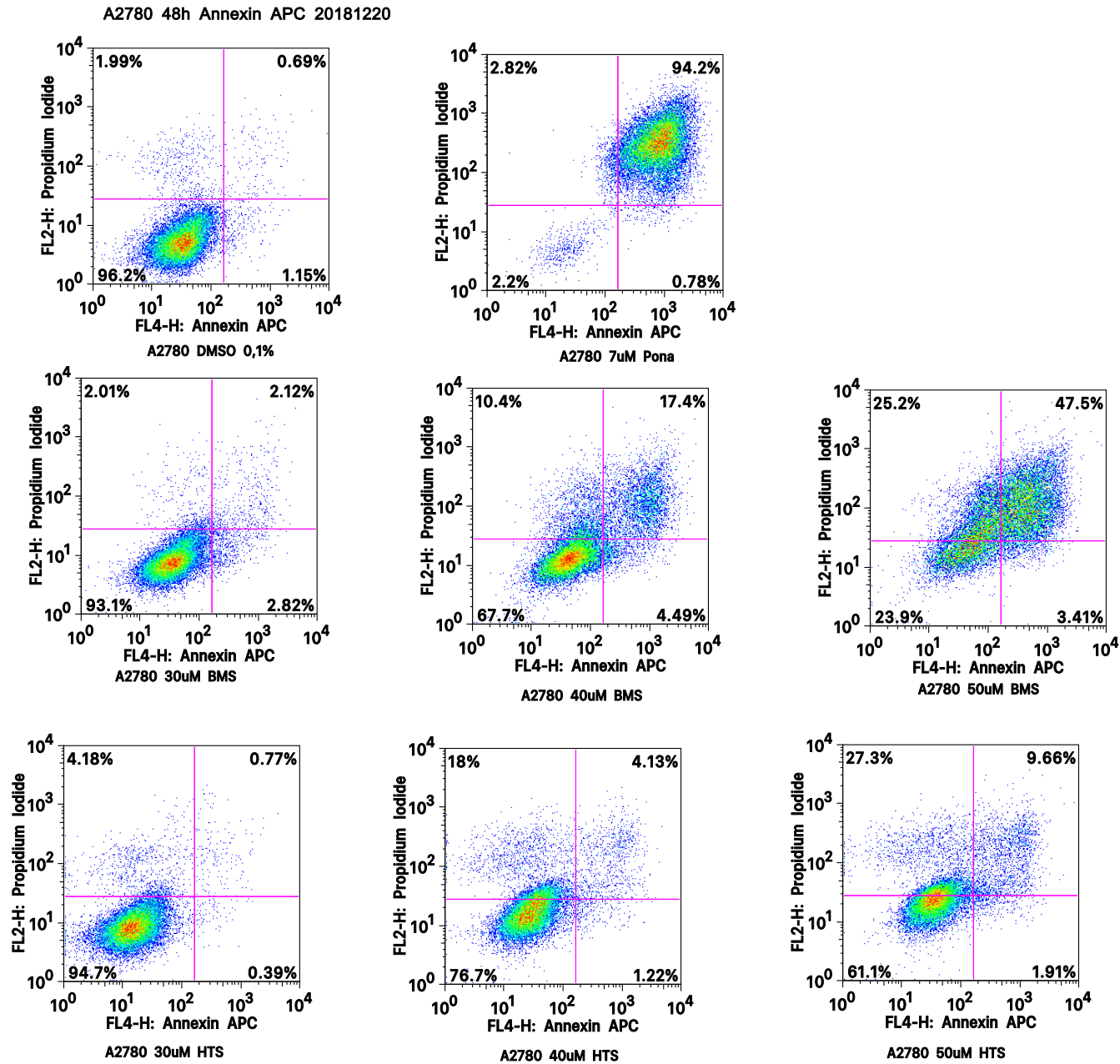

Supplementary Figure S5A

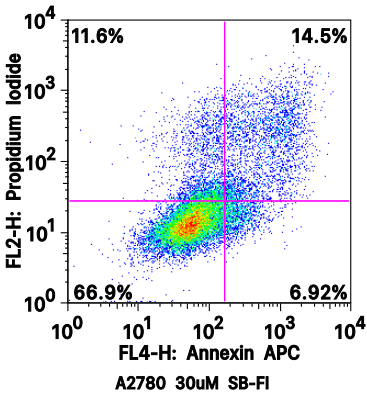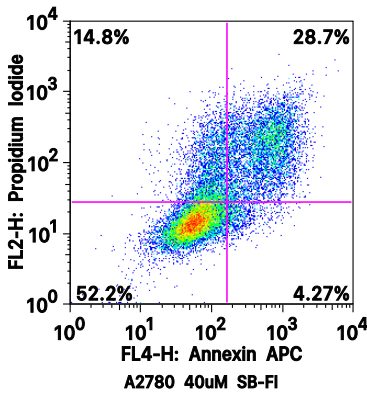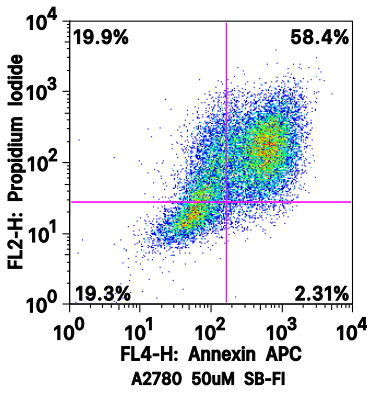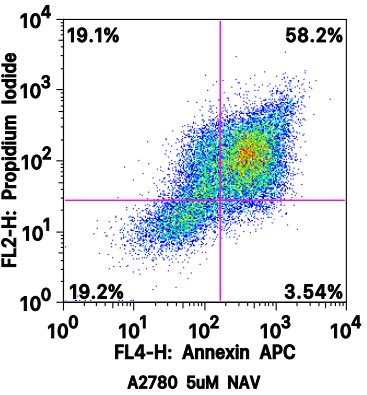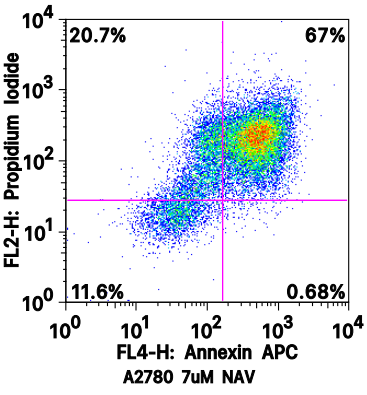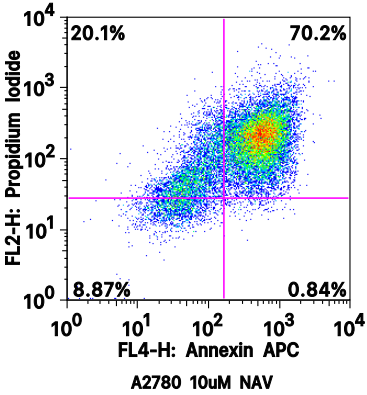

Supplement: Supplementary file 1 [file cancers-14-06004-s001.zip › Supplementary Figure S5A.pdf]

20181221 A2780 48h Caspase 3

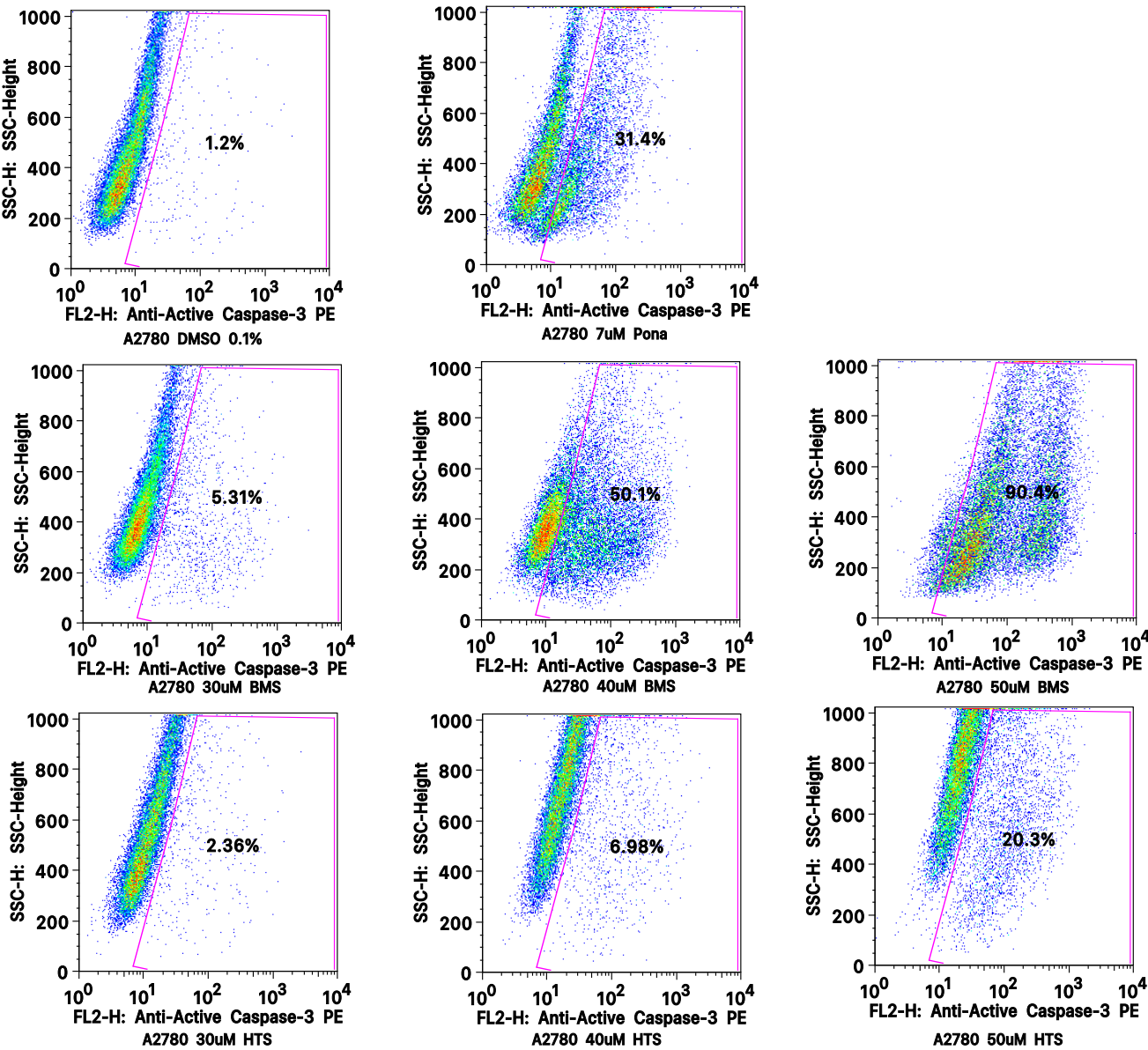

Supplementary Figure S5B

Layout

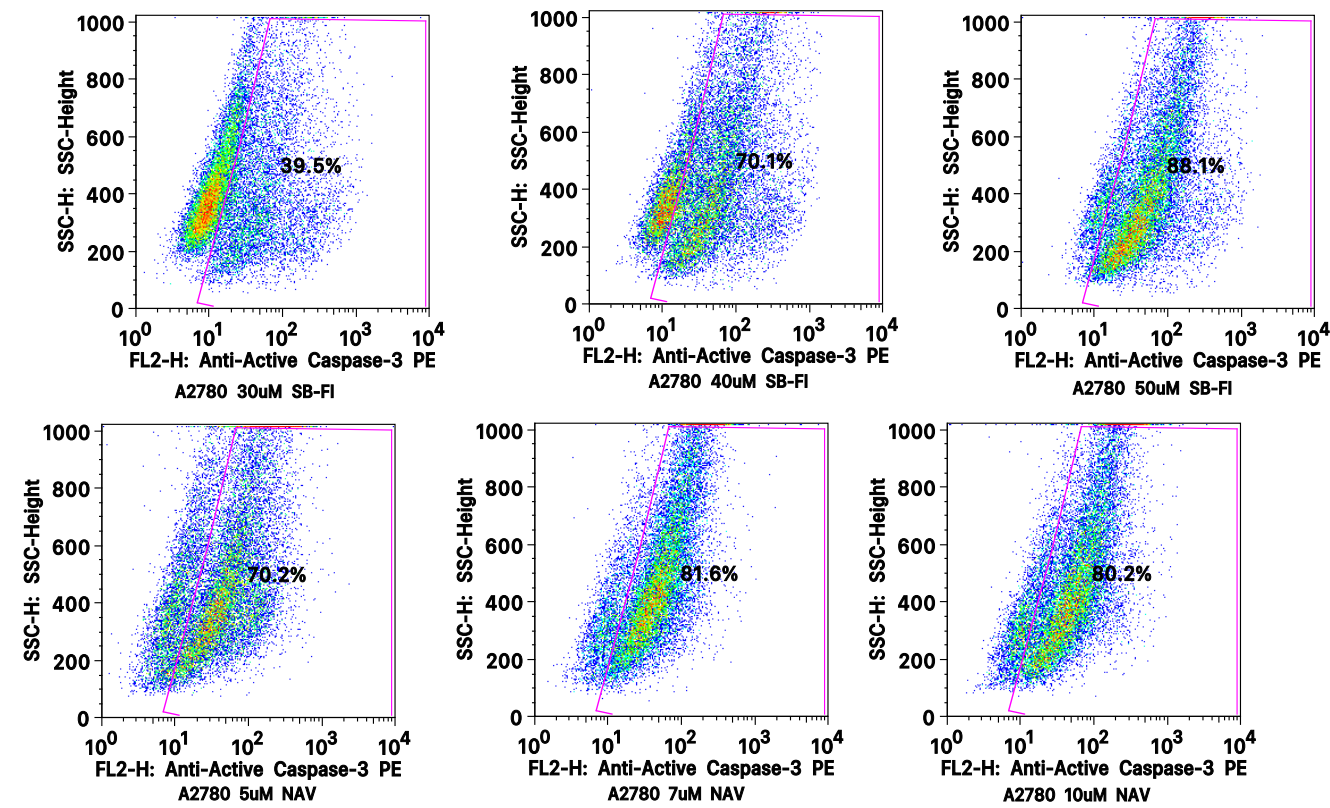

Supplement: Supplementary file 1 [file cancers-14-06004-s001.zip › Supplementary Figure S5B.pdf]

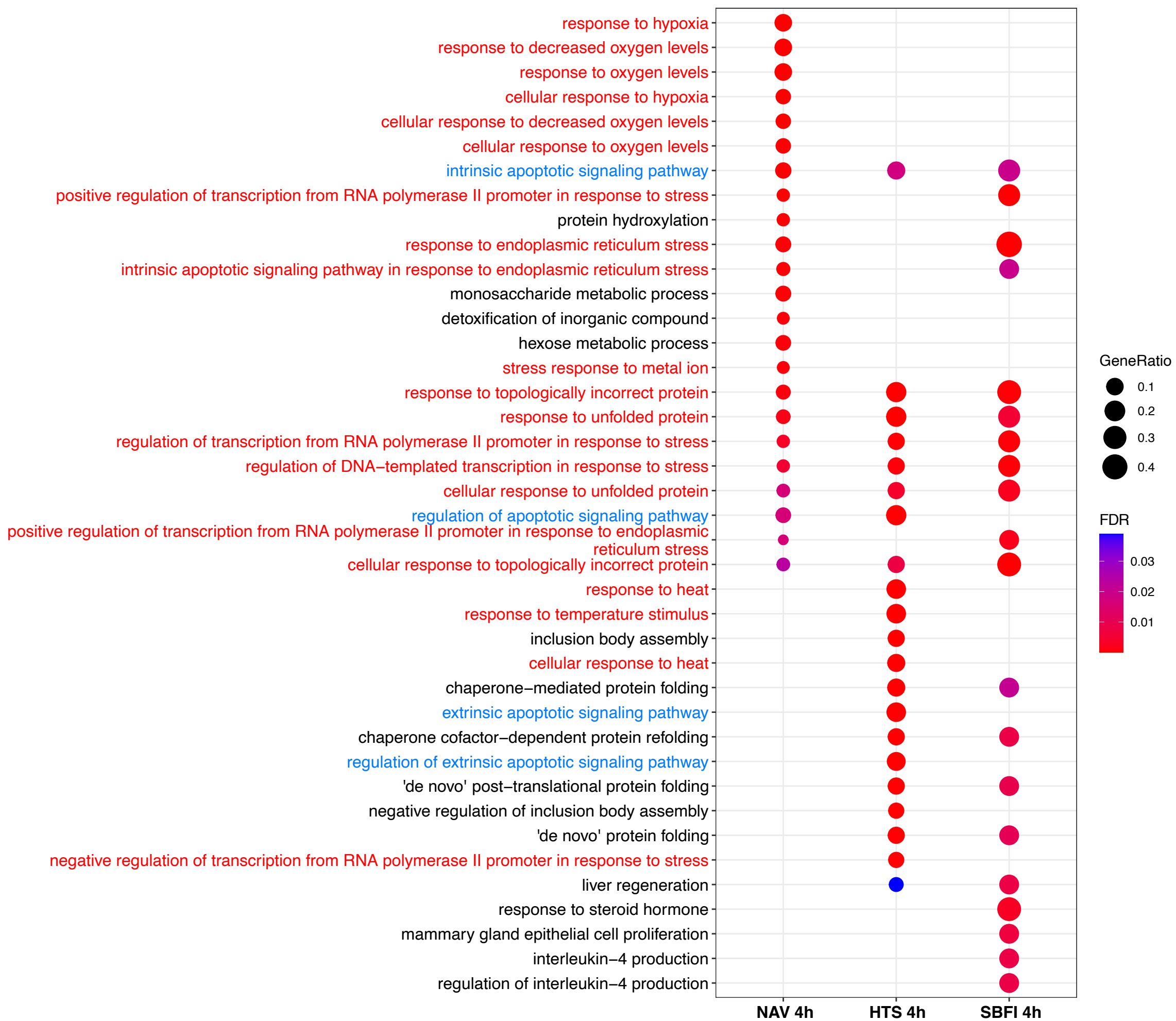

Supplementary Figure S6

Supplement: Supplementary file 1 [file cancers-14-06004-s001.zip › Supplementary Figure S6.pdf]

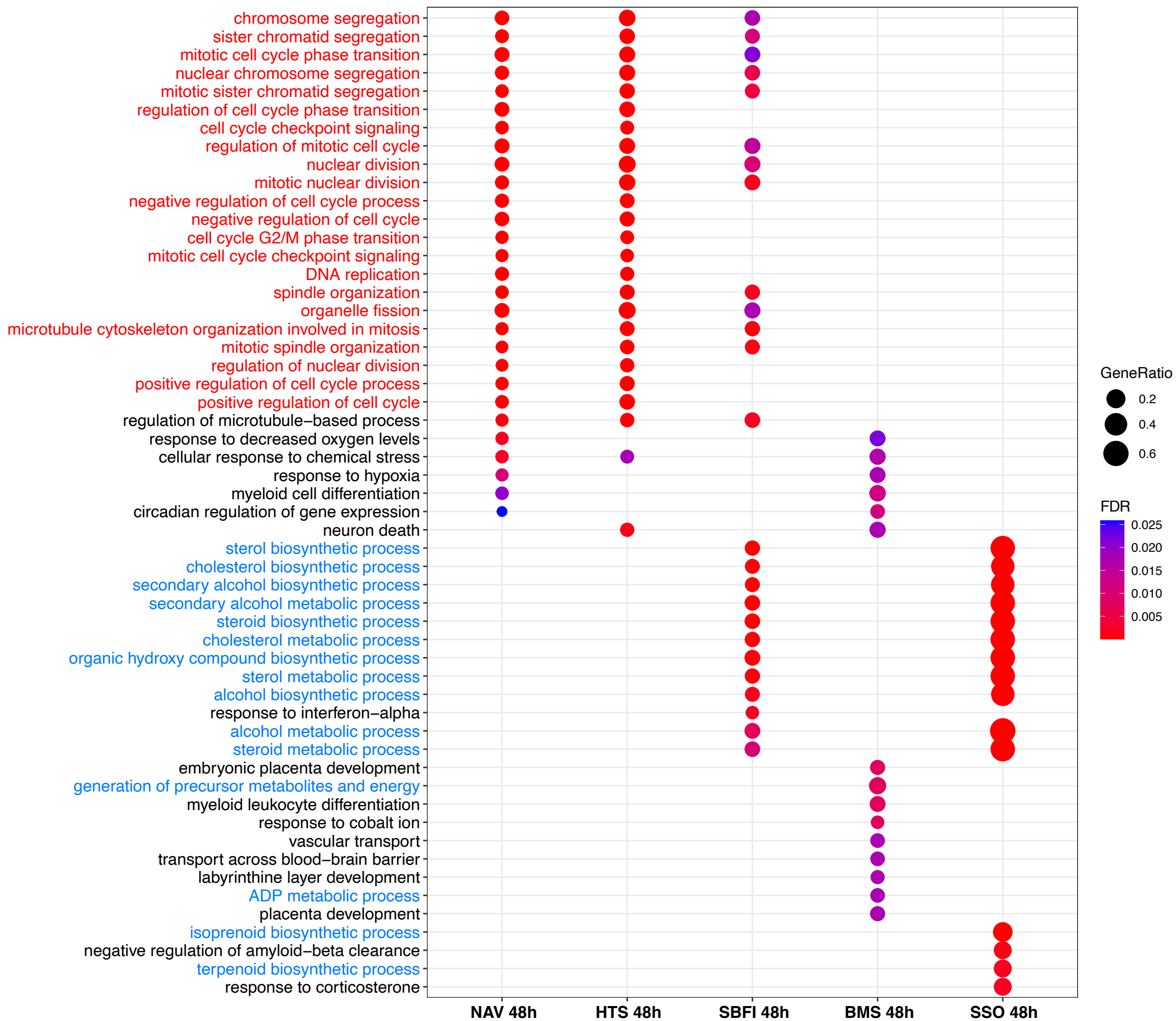

Supplementary Figure S7

Supplement: Supplementary file 1 [file cancers-14-06004-s001.zip › Supplementary Figure S7.pdf]

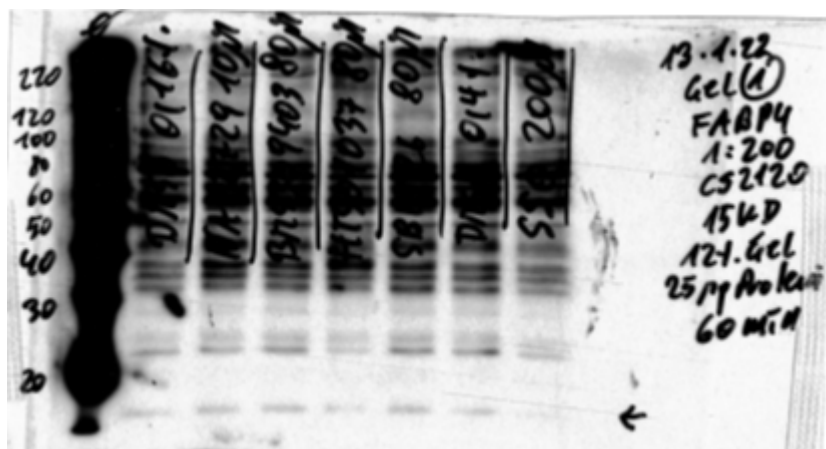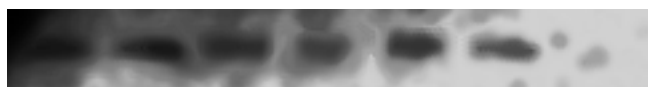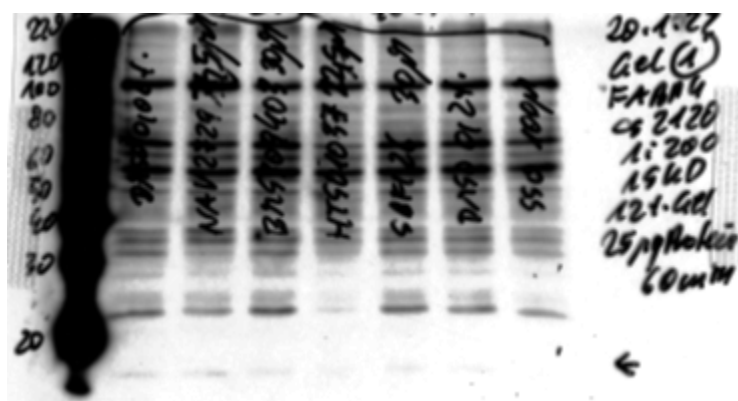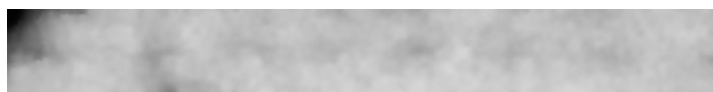

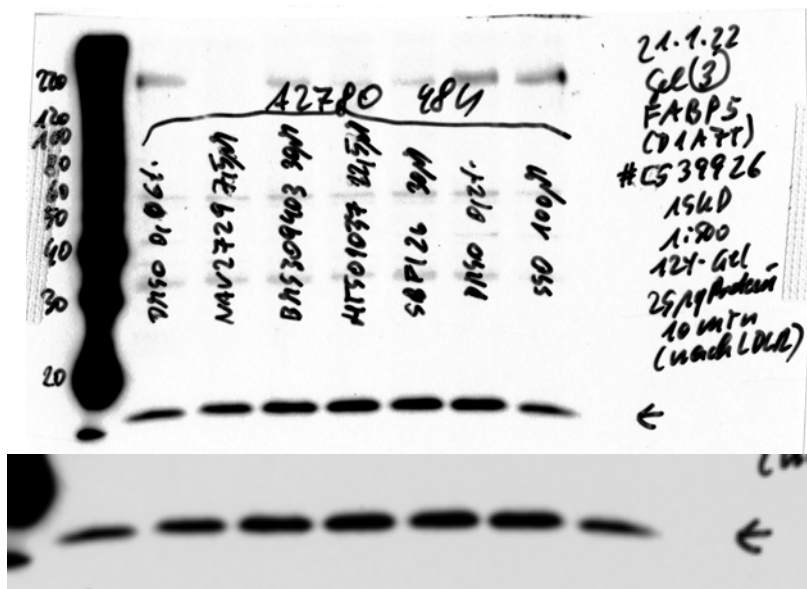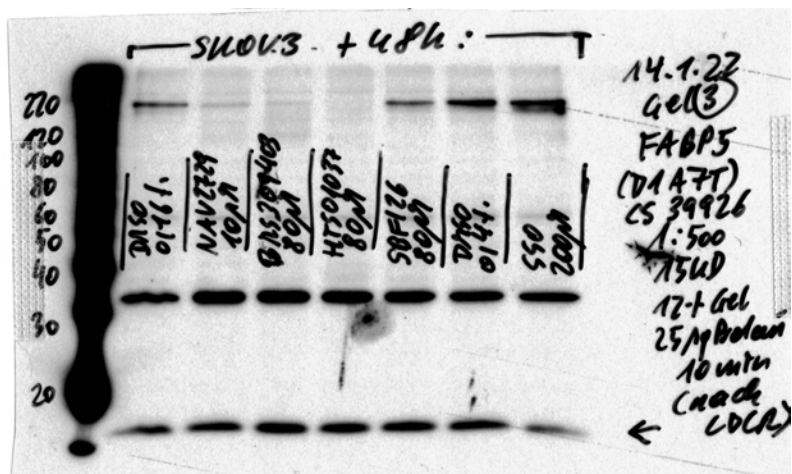

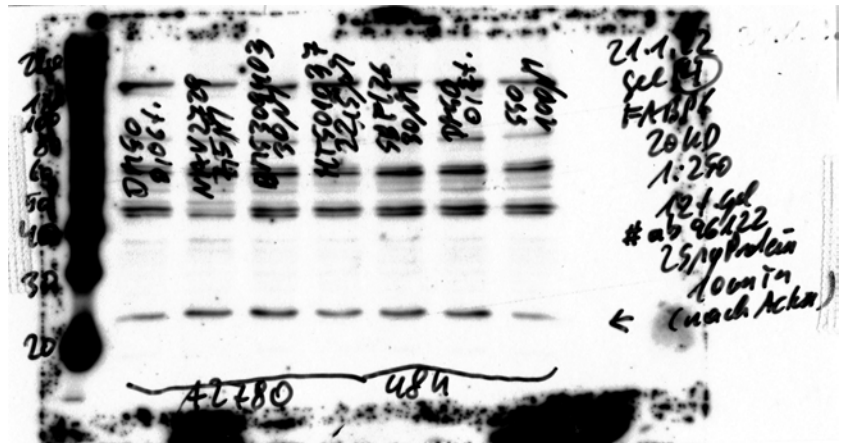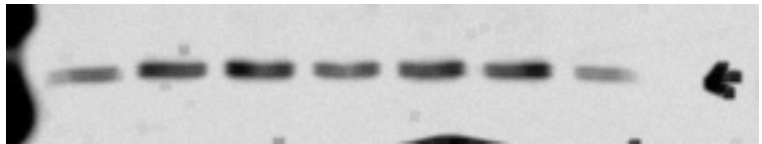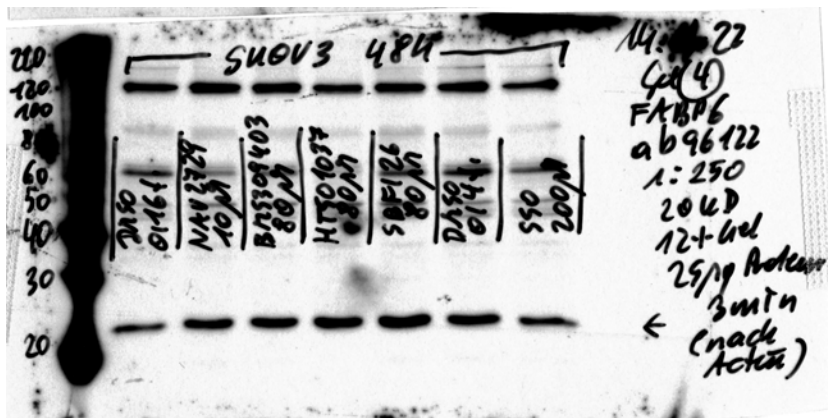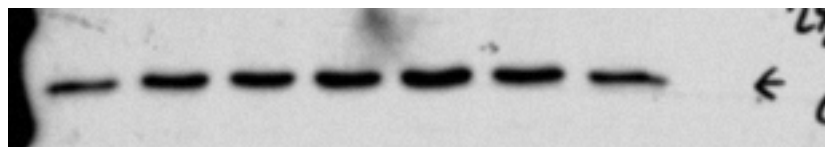

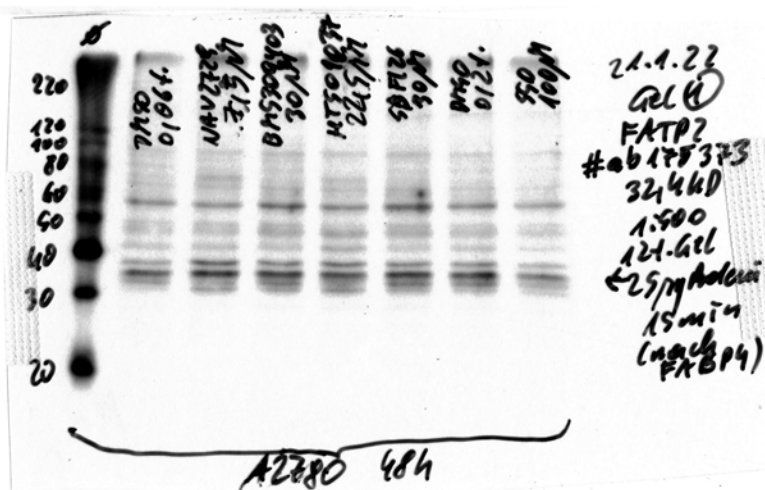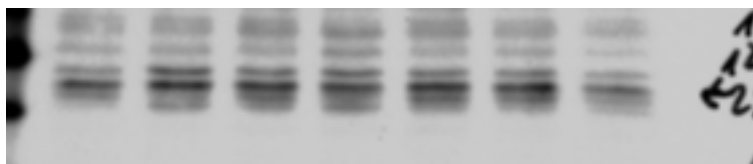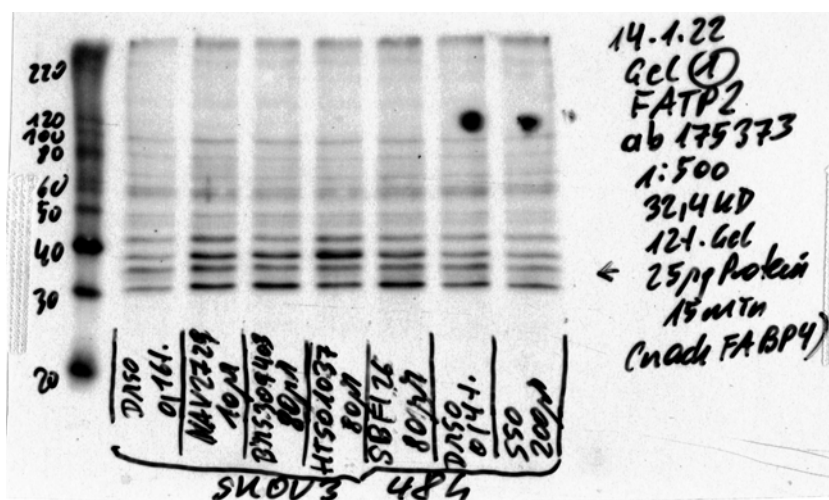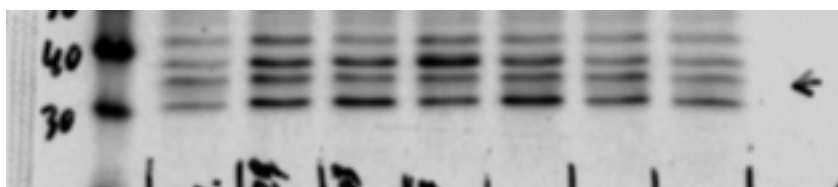

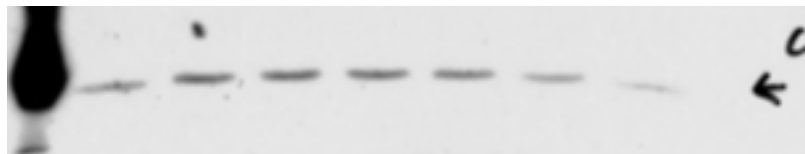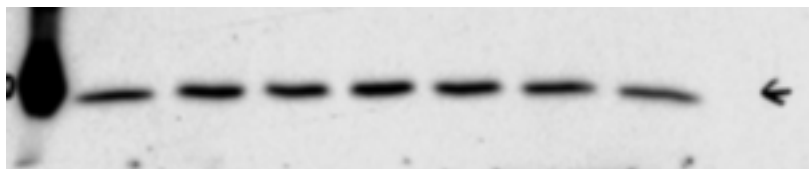

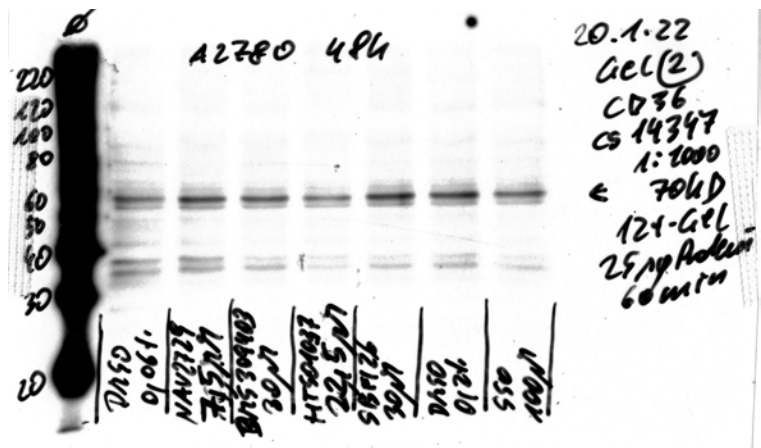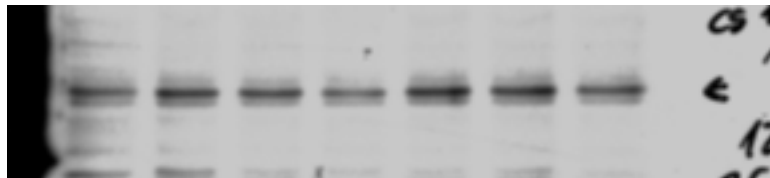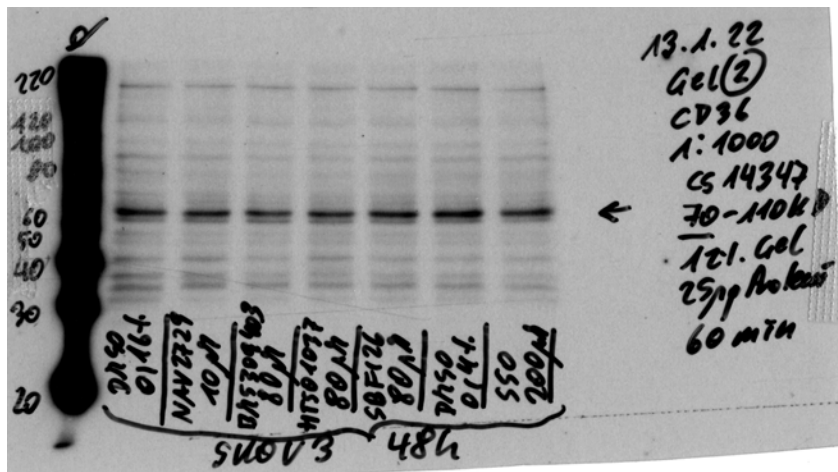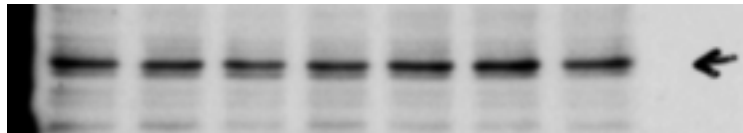

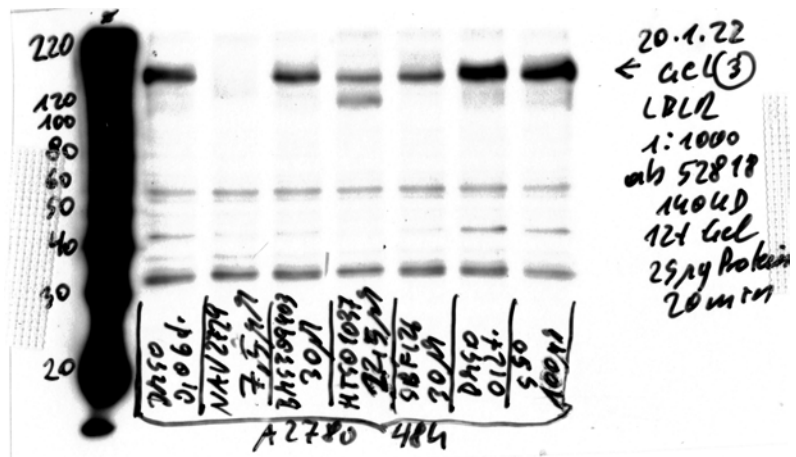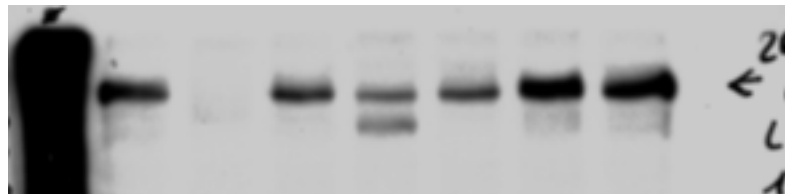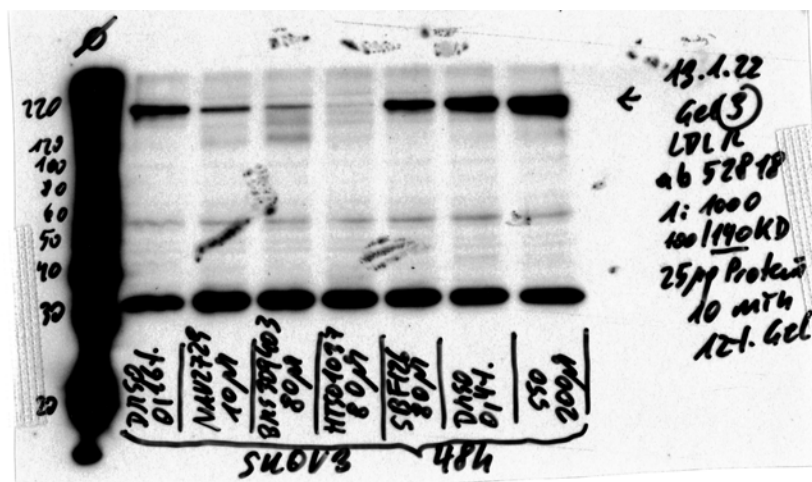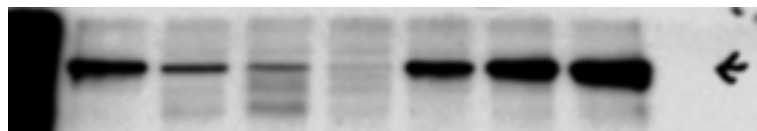

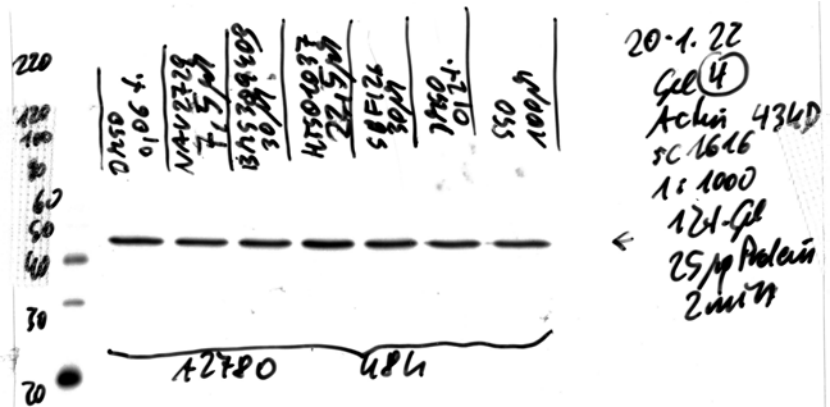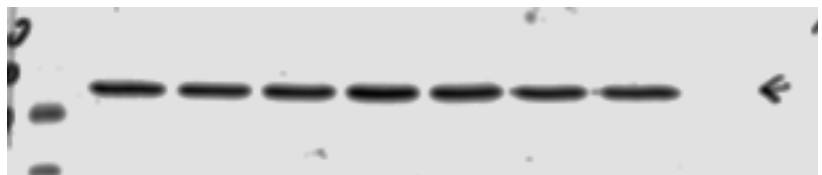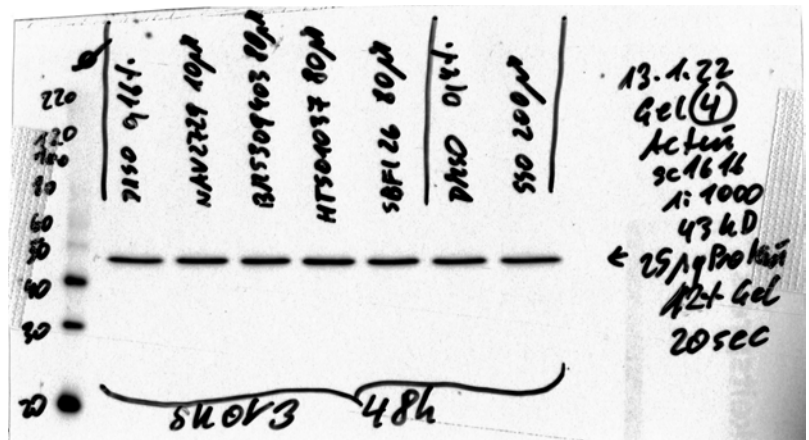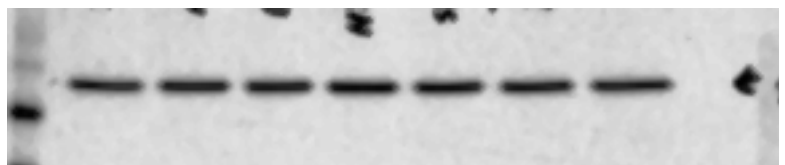

Supplement: Supplementary file 1 [file cancers-14-06004-s001.zip › Supplementary Figure S8.pdf]
